# The Degradation of Aqueous Oxytetracycline by an O_3_/CaO_2_ System in the Presence of ${\mathrm{HCO}}_{3}^{-}$: Performance, Mechanism, Degradation Pathways, and Toxicity Evaluation

**DOI:** 10.3390/molecules29030659

**Published:** 2024-01-31

**Authors:** Zedian Li, Liangrui Xiang, Shijia Pan, Dahai Zhu, Shen Li, He Guo

**Affiliations:** 1 School of Energy and Materials, Shanghai Polytechnic University, Shanghai 201209, China; lzd111303@outlook.com (Z.L.); zhudh@sspu.edu.cn (D.Z.); 2 College of Biology and the Environment, Nanjing Forestry University, Nanjing 210037, China; 17755053066@163.com (L.X.); 13675128810@163.com (S.P.); 3 Anhui Jiuwu Tianhong Environmental Protection Technology Co., Ltd., Hefei 230011, China; lishen3569@outlook.com

**Keywords:** O_3_, CaO_2_, ${\mathrm{HCO}}_{3}^{-}$, oxytetracycline, degradation

## Abstract

This research constructed a novel O_3_/CaO_2_/${\mathrm{HCO}}_{3}^{-}$ system to degrade antibiotic oxytetracycline (OTC) in water. The results indicated that CaO_2_ and ${\mathrm{HCO}}_{3}^{-}$ addition could promote OTC degradation in an O_3_ system. There is an optimal dosage of CaO_2_ (0.05 g/L) and ${\mathrm{HCO}}_{3}^{-}$ (2.25 mmol/L) that promotes OTC degradation. After 30 min of treatment, approximately 91.5% of the OTC molecules were eliminated in the O_3_/CaO_2_/${\mathrm{HCO}}_{3}^{-}$ system. A higher O_3_ concentration, alkaline condition, and lower OTC concentration were conducive to OTC decomposition. Active substances including ·OH, ^1^O_2_, $\cdot O_{2}^{-}$, and ·${\mathrm{HCO}}_{3}^{-}$ play certain roles in OTC degradation. The production of ·OH followed the order: O_3_/CaO_2_/${\mathrm{HCO}}_{3}^{-}$ > O_3_/CaO_2_ > O_3_. Compared to the sole O_3_ system, TOC and COD were easier to remove in the O_3_/CaO_2_/${\mathrm{HCO}}_{3}^{-}$ system. Based on DFT and LC-MS, active species dominant in the degradation pathways of OTC were proposed. Then, an evaluation of the toxic changes in intermediates during OTC degradation was carried out. The feasibility of O_3_/CaO_2_/${\mathrm{HCO}}_{3}^{-}$ for the treatment of other substances, such as bisphenol A, tetracycline, and actual wastewater, was investigated. Finally, the energy efficiency of the O_3_/CaO_2_/${\mathrm{HCO}}_{3}^{-}$ system was calculated and compared with other mainstream processes of OTC degradation. The O_3_/CaO_2_/${\mathrm{HCO}}_{3}^{-}$ system may be considered as an efficient and economical approach for antibiotic destruction.

## 1. Introduction

In recent decades, antibiotics have been widely used in the pharmaceutical and animal husbandry industries. While providing substantial social benefits, they inevitably cause serious environmental pollution [1,2]. Oxytetracycline (OTC) is a kind of tetracycline antibiotic [3] with broad-spectrum bacteriostasis and a low price. As a veterinary drug, it is widely used in animal husbandry worldwide [4]. However, excessive use of OTC cannot be fully absorbed by animals, and there will be a large amount of OTC in the environment [5,6]. A residual concentration of OTC in water is still present after treatment by sewage treatment plants. A high concentration of OTC in water will slow down the development of fish [7] and inhibit photosynthesis in plants [8]. The naphthalene ring structure has antibacterial properties and chemical stability, which makes it difficult to remove OTC through traditional water treatment processes [9]. Therefore, it is imperative to seek an effective method to remove OTC in water.

Oxidation is an essential reaction from an industrial and academic point of view. In general, selecting oxidants is significant for heterogeneous reaction systems. An advanced oxidation process (AOP_S_) is recognized as an effective method for treating wastewater with potential environmental risks. One of the most common active species in AOP_S_ is hydroxyl radicals (·OH), which have strong oxidation properties and can convert pollutants into carbon dioxide and water. A simple and commonly used substance in AOPs is O_3_ [10]. O_3_ is considered to be a clean oxidant in which organic pollutants are oxidized directly or indirectly with free radicals generated by O_3_ to achieve the degradation of organic pollutants [11]. The direct oxidation reaction time is longer, the oxidation rate is slow, and it has a specific selectivity. Indirect oxidation refers to the solid oxidizing free radicals (mainly ·OH) generated by the catalytic O_3_ reaction, which can quickly decompose most organic pollutants in water. In the indirect oxidation method, the use of a catalyst to activate O_3_ increases the oxidation capacity of O_3_ and promotes the degradation of organic pollutants. Recently, the couple of O_3_ and other oxidation processes has become very popular, such as H_2_O_2_/O_3_ [12], ultrasonic treatment ozonation [13], and photocatalytic ozonation [14]. Among them, the most effective method of O_3_ indirect oxidation technology is the O_3_/H_2_O_2_ method, which is also the simplest method for ·OH [15]. However, H_2_O_2_ is relatively dangerous, with the characteristic of being flammable and explosive [16].

So far, many studies have confirmed that a combination of several treatment technologies can improve the degradation and mineralization of organic pollutants in wastewater [17]. Some catalysts can promote the better degradation of pollutants by O_3_. As a substitute for liquid hydrogen peroxide, calcium peroxide (CaO_2_), a new solid oxidant, has attracted a lot of attention in the environmental field due to its remarkable advantages, such as a high efficiency, safety, stability, controllability, and low cost [18,19]. The process of releasing H_2_O_2_ from calcium peroxide is slow, which can be controlled by adjusting the pH, which avoids the disadvantage of wasting oxygen and the short action time caused by the rapid decomposition of liquid H_2_O_2_. For some pollutants (such as PPCP and PAH), the removal effect of solid calcium peroxide will exceed that of liquid H_2_O_2_ [20,21]. The O_3_/H_2_O_2_ system created by O_2_/CaO_2_ has a low oxidation rate and poor selectivity in the absence of an activator. Sodium bicarbonate was added to the system as an auxiliary catalyst [22]. On one hand, ${\mathrm{HCO}}_{3}^{-}$ reacts with H_2_O_2_ to generate peroxymonocarbonate (${\mathrm{HCO}}_{4}^{-}$), which continuously activates H_2_O_2_ and converts it into highly active free radicals such as ·O_2_^−^, singlet oxygen (^1^O_2_), and so on [23]. On the other hand, ${\mathrm{HCO}}_{3}^{-}$ is a recognized promoter of O_3_ decomposition, promoting the efficient oxidation of O_3_ into ·CO_3_^−^ and the conversion of O_3_ into indirect oxidation [24]. O_3_/CaO_2_/${\mathrm{HCO}}_{3}^{-}$ innovatively proposes a safe and fast O_3_/H_2_O_2_ system. Meanwhile, the addition of ${\mathrm{HCO}}_{3}^{-}$ could activate H_2_O_2_ and O_3_ and significantly improve the oxidation capacity of the system [25]. As a whole, the advantages of the O_3_/CaO_2_/${\mathrm{HCO}}_{3}^{-}$ system can be listed as: firstly, the synergy effect between O_3_ and CaO_2_ can promote the generation of ·OH. Secondly, the OH^−^ released by CaO_2_ will form an alkaline environment, which will further promote the transformation of O_3_ into ·OH. Thirdly, ${\mathrm{HCO}}_{3}^{-}$ activates O_3_ radicals and catalyzes the production of ·OH radicals. Finally, the decomposition products of CaO_2_/${\mathrm{HCO}}_{3}^{-}$ are non-toxic and harmless. However, as far as is known, there have been few reports that O_3_ activates CaO_2_/${\mathrm{HCO}}_{3}^{-}$ for antibiotic OTC degradation, and its synergistic mechanism is still unclear.

Therefore, the O_3_/CaO_2_/${\mathrm{HCO}}_{3}^{-}$ system was constructed for the efficient degradation of OTC in this paper. The influence of the effect of CaO_2_ dosage, ${\mathrm{HCO}}_{3}^{-}$ dosage, O_3_ concentration, initial pH value, and OTC initial concentration on OTC degradation was investigated. The role of active substances in the OTC decomposition system was inspected via the capture agent experiment. The generated active species were characterized using chemical methods and electron spin resonance (ESR). The degradation mechanism and process were examined. The toxicity of OTC and intermediates was analyzed, as was the feasibility of the O_3_/CaO_2_/${\mathrm{HCO}}_{3}^{-}$ system for the treatment of other antibiotics and actual wastewater. Finally, the energy efficiency of the system was compared with that of other technology.

## 2. Results and Discussion

### 2.1. Effect of CaO_2_

**Figure 1:**
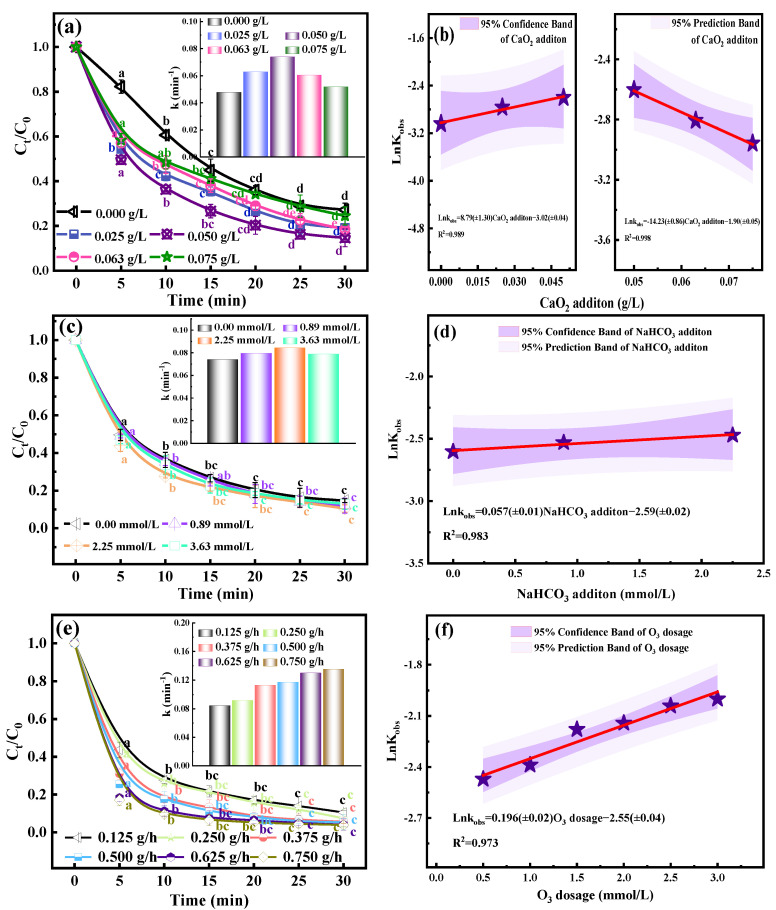
Impact of CaO_2_ addition: (**a**) degradation efficiency and (**b**) linear fitting of dynamic constants; impact of NaHCO_3_ addition: (**c**) degradation efficiency and (**d**) linear fitting of dynamic constants; impact of O_3_ dosage: (**e**) degradation efficiency and (**f**) linear fitting of dynamic constants. Experimental conditions: [OTC] = 80 mg/L, 400 mL, 32 mg.

Figure 1a,b reveal the changes in the OTC degradation efficiency under different CaO_2_ additions. The lowercase letters in the picture represent the difference between the data, and the star symbol represents the value of lnK_obs_. Experiments were carried out with fixed OTC concentrations at different CaO_2_ concentrations. It was observed that the O_3_/CaO_2_ system had a significant effect on the degradation of OTC within 30 min. When the dosage of CaO_2_ was 0.050 g/L, the degradation efficiency of OTC could reach 85.3%, which was 23.2% higher than that of the sole O_3_ system. The degradation efficiency (slope of degradation curve) of OTC increased with an increase in the CaO_2_ concentration, which indicates that CaO_2_ is conducive to the degradation of OTC. It is worth noting that, when the dosage of CaO_2_ was higher than the optimal dosage (0.050 g/L), the degradation efficiency gradually slowed down. The first-order kinetic constant in the upper right corner of Figure 1a can also reflect this trend.

CaO_2_ is a good solid source of H_2_O_2_ [26]. It can be regularly converted into H_2_O_2_ and O_2_ when dissolved in water (Equations (1) and (2)) [27]. The H_2_O_2_ it releases can reduce disproportionation and maintain the reaction for a longer time. H_2_O_2_ will cause O_3_ (E° = 2.07 V/NHE) to decompose and transform into non-selective ·OH (E° = 2.80 V/NHE) [28]. The formation of Ca(OH)_2_ will affect the pH value of the solution. The alkaline environment brought about makes H_2_O_2_ decompose into ${\mathrm{HO}}_{2}^{-}$. ${\mathrm{HO}}_{2}^{-}$ is converted into ·OH through a series of reactions with O_3_ (Equations (3)–(13)) [29,30,31,32,33]. This ·OH is a strong oxidant, which can effectively promote the degradation of OTC. However, when the concentration of CaO_2_ is too high, too much H_2_O_2_ will be produced [34]. Excessive H_2_O_2_ will decompose by itself and need to consume active free radicals such as ·OH (Equations (14)–(16)), which will adversely affect the degradation of OTC [35]. The oxidation potentials of different free radicals are displayed in Table S1.

(1) $${\mathrm{CaO}}_{2}{+2H}_{2}O\;\to \;H_{2}O_{2}{+\mathrm{Ca}(\mathrm{OH})}_{2}$$

(2) $$H_{2}O_{2}{+2O}_{3}\;\to {\;2\mathrm{OH}+3O}_{2}$$

(3) $${\mathrm{OH}}^{-}{+O}_{3}\;\to {\;\mathrm{HO}}_{2}^{-}{+O}_{2}$$

(4) $${\mathrm{HO}}_{2}^{-}{+O}_{3}\;\to {\;\mathrm{HO}}_{2}{+O}_{3}^{-}$$

(5) $${\mathrm{HO}}_{2}^{-}\;\to {\;\cdot O}_{2}^{-}+\cdot H$$

(6) $${\cdot O}_{3}^{-}{+H}^{+}\;\to {\;\cdot \mathrm{HO}}_{3}$$

(7) $${\cdot \mathrm{HO}}_{3}\;\to {\;O}_{2}+\cdot \mathrm{OH}$$

(8) $${\cdot O}_{3}^{-}{+O}_{3}\to {\;O}_{2}{+\cdot O}_{3}^{-}$$

(9) $${\mathrm{HO}}_{2}^{-}{+O}_{3}\to {\;\mathrm{HO}}_{5}^{-}$$

(10) $${\mathrm{HO}}_{5}^{-}\;\to {\;\cdot \mathrm{HO}}_{2}{+\cdot O}_{3}^{-}$$

(11) $${\mathrm{HO}}_{5}^{-}\;\to {\;2O}_{2}{+\mathrm{OH}}^{-}$$

(12) $${\cdot O}_{3}^{-}\;\to {\;\cdot O}^{-}{+O}_{2}$$

(13) $${\cdot O}^{-}{+H}_{2}O\;\to {\;\cdot \mathrm{OH}+\mathrm{OH}}^{-}$$

(14) $${2H}_{2}O_{2}\;\to {\;O}_{2}{+2H}_{2}O$$

(15) $$H_{2}O_{2}+\cdot \mathrm{OH}\;\to {\;\cdot \mathrm{HO}}_{2}{+H}_{2}O$$

(16) $${\mathrm{HO}}_{2}^{-}+\cdot \mathrm{OH}\;\to {\;\cdot \mathrm{HO}}_{2}{+\mathrm{OH}}^{-}$$

### 2.2. Effect of ${HCO}_{3}^{-}$ Dosage

The degradation of OTC with time under the conditions of different ${\mathrm{HCO}}_{3}^{-}$ additions is shown in Figure 1c,d. To explore the CaO_2_/${\mathrm{HCO}}_{3}^{-}$ combined process, with the addition of ${\mathrm{HCO}}_{3}^{-}$ as a variable, the experiment was carried out with the optimal CaO_2_ concentration and fixed OTC concentration. It was observed that the O_3_/CaO_2_/${\mathrm{HCO}}_{3}^{-}$ system had a further improvement in the degradation of OTC compared to O_3_/CaO_2_ within the reaction time of 30 min. When the dosage of ${\mathrm{HCO}}_{3}^{-}$ was 2.25 mmol/L, the degradation efficiency of OTC could reach 91.5%, which was 7.3% higher than that of the O_3_/CaO_2_ system and 32.1% higher than that of the sole O_3_ system. The degradation efficiency of OTC increased with an increase in the ${\mathrm{HCO}}_{3}^{-}$ concentration. Further increasing the concentration of ${\mathrm{HCO}}_{3}^{-}$ based on the optimal dosage of ${\mathrm{HCO}}_{3}^{-}$ will inhibit the degradation of OTC. Similarly, the first-order kinetic constant in the upper right corner of Figure 1c also reflects this trend.

${\mathrm{HCO}}_{3}^{-}$ reacts with H^+^ in water to produce CO_2_, H_2_O_2_ reacts with OH^−^ in water to produce OOH^−^, and CO_2_ reacts with OOH^−^ to produce ${\mathrm{HCO}}_{4}^{-}$. The active free radicals released by ${\mathrm{HCO}}_{4}^{-}$ (${\cdot \mathrm{CO}}_{3}^{-}$) can activate H_2_O_2_ to generate ·HO_2_, and then react to generate ${\cdot O}_{2}^{-}$, ^1^O_2_ and other oxygen-active substances (Equations (17)–(22)) [36,37]. The consumption of ${\cdot \mathrm{CO}}_{3}^{-}$ for activating H_2_O_2_ can be regenerated into ${\cdot \mathrm{CO}}_{3}^{-}$ through intermediate ${\mathrm{HCO}}_{3}^{-}$ to form a cycle, which is the synergistic effect of ${\mathrm{HCO}}_{3}^{-}$ and CaO_2_ ((Equation (19)) [23,38].

(17) $${\mathrm{HCO}}_{3}^{-}{+H}_{2}O_{2}\;\to {\;\mathrm{HCO}}_{4}^{-}{+H}_{2}O$$

(18) $${\mathrm{HCO}}_{4}^{-}\;\to {\;\cdot \mathrm{CO}}_{3}^{-}+\cdot \mathrm{OH}$$

(19) $${\mathrm{HCO}}_{3}^{-}\;+\;\cdot \mathrm{OH}\to \;{\mathrm{CO}}_{3}^{-}\;+\;H_{2}O$$

(20) $$H_{2}O_{2}{+\cdot \mathrm{CO}}_{3}^{-}\;\to {\;\mathrm{HCO}}_{3}^{-}{+\cdot \mathrm{HO}}_{2}$$

(21) $$\cdot {\mathrm{HO}}_{2}\;\to \;H^{+}\;+\;{\cdot O}_{2}^{-}$$

(22) O2−+·OH → O12+OH−

The synergistic effect between ${\mathrm{HCO}}_{3}^{-}$ and O_3_ is mainly reflected in: OH^−^ and ${\cdot O}_{2}^{-}$ promote the decomposition of O_3_ into ${\cdot O}_{3}^{-}$, ·$O_{3}^{-}$, through a chain reaction, generates more ${\cdot O}_{2}^{-}$, ${\cdot O}_{2}^{-}$, in turn, promotes the decomposition of O_3_ (Equations (23)–(31)) [39,40,41]. There is a balance point between the generation of ${\cdot O}_{2}^{-}$ and the decomposition of O_3_, which also explains the phenomenon that the degradation efficiency of OTC decreases when the ${\mathrm{HCO}}_{3}^{-}$ dosing amount is higher than the optimal amount [42].

(23) $$O_{3}{+\cdot O}_{2}^{-}\;\to {\;\cdot O}_{3}^{-}{+O}_{2}$$

(24) $$O_{3}\;+\;{\mathrm{OH}}^{-}\;\to \;HO_{2}^{-}O_{2}$$

(25) $$O_{3}\;+\;HO_{2}^{-}\;\to \;\cdot O_{3}^{-}\;+\;\cdot {\mathrm{HO}}_{2}$$

(26) $${\cdot O}_{3}^{-}{+H}^{+}\;\to {\;\cdot \mathrm{HO}}_{3}$$

(27) $${\cdot \mathrm{HO}}_{3}\;\to {\;\cdot \mathrm{OH}+O}_{2}$$

(28) $$O_{3}\;+\;\cdot \mathrm{OH}\;\to \;{\cdot \mathrm{HO}}_{2}\;+\;O_{2}$$

(29) $$H_{2}O_{2}+\cdot \mathrm{OH}\;\to {\;\cdot \mathrm{HO}}_{2}\;+\;H_{2}O$$

(30) $${\mathrm{HO}}_{2^{-}}+\cdot \mathrm{OH}\;\to {\;\cdot O}_{2}^{-}{+H}_{2}O\;$$

(31) $$\cdot HO_{2}\;\to \;\cdot O_{2}^{-}\;+\;H^{+}$$

### 2.3. Effect of O_3_ Dosage

The degradation of OTC under the conditions of different O_3_ concentrations is presented in Figure 1e,f. As the amount of O_3_ increased from 0.125 g/h to 0.75 g/h, the OTC degradation efficiency increased significantly from 91.5% to 97.2%, and the reaction time when the OTC degradation efficiency reached 90% was shortened from 30 min to 5 min. The reasons were as follows: firstly, the larger O_3_ flux increased the contact area between the gas phase and the liquid phase, and the number of effective molecular collisions increased, which improved the reaction rate. Secondly, according to Henry’s Law, the more significant the O_3_ concentration, that is, the increase in X_Z_ per unit volume, the more increased the pressure of the O_3_ gas, thus promoting the mass transfer rate of O_3_ between the two phases [43]. Third, the O_3_ concentration will affect the concentrations of H_2_O_2_ and ·OH in the reaction system. A higher concentration will promote the generation of more active free radicals and affect the degradation effect [44]. However, a high-concentration O_3_ treatment process means a high cost, which will affect the economy of this process.

### 2.4. Effect pH Value

**Figure 2:**
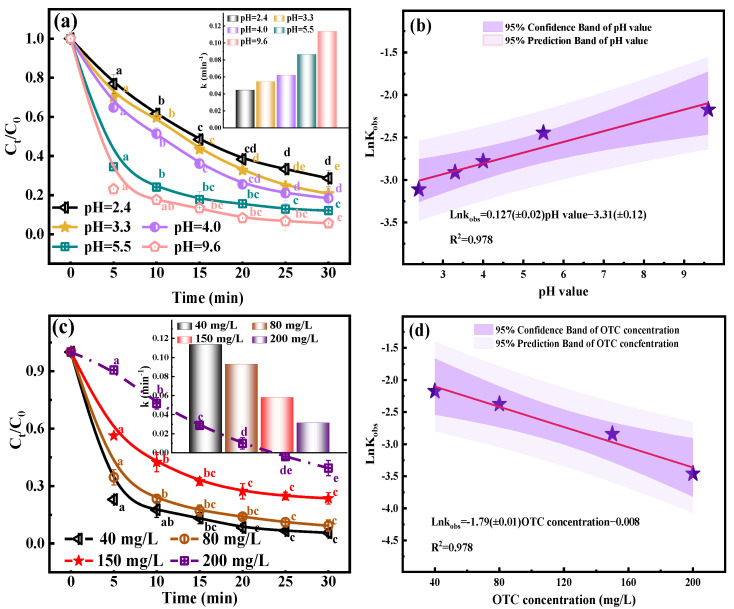
Impact of pH value: (**a**) degradation efficiency; (**b**) linear fitting of dynamic constants, impact of OTC concentration; (**c**) degradation efficiency; and (**d**) linear fitting of dynamic constants. Experimental conditions: [CaO_2_] = 0.05 g/L, [O_3_] = 0.75 g/h, [${\mathrm{HCO}}_{3}^{-}$] = 2.25 mmol/L.

The degradation of OTC with time under the conditions of different initial pHs is shown in Figure 2a. With an increase in the initial pH of the solution, the environment changed from acidic to neutral and then to alkaline. The OTC degradation efficiency was enhanced from 71.4% to 94.4%. This phenomenon was mainly due to the direct oxidation of O_3_ molecules with pollutants in an acidic environment. In an alkaline environment, the concentration of dissolved O_3_ decreases [45], forcing O_3_ molecules to be activated into active free radicals such as ·OH and ${\cdot O}_{2}^{-}$ (Equations (24)–(32)) to react with OTC [46]. Because the indirect reaction is dominant, the oxidation potential of ·OH is higher than that of O_3_ molecules, and the oxidation ability is stronger, which is more conducive to the degradation of OTC. On the other hand, an alkaline environment is more conducive to the release of H_2_O_2_ from CaO_2_ and the self-decomposition of H_2_O_2_, which will produce more active free radicals and promote the degradation of pollutants [47]. Similarly, the O_3_ treatment process under alkaline conditions also means an increase in cost, and the natural water environment is mainly neutral or weakly alkaline, so neutral environmental conditions will continue to be used in subsequent studies.

### 2.5. Effect OTC Concentration

The degradation of OTC with time under the conditions of different OTC concentrations is shown in Figure 2c. As the initial OTC concentration was raised from 40 mg/L to 200 mg/L, the OTC degradation efficiency decreased significantly, the degradation efficiency decreased from 94.4% to 60.6%, and the kinetic constant decreased from 0.114 min^−1^ to 0.031 min^−1^ (inset Figure 2c). It is unreasonable to judge the OTC degradation only by the initial OTC concentration. We calculated the total removal of the four initial OTC concentrations in Figure 2c, which were 15.1 mg, 29.0 mg, 45.8 mg, and 48.5 mg, respectively. Comparing the kinetic constant and total removal amount of OTC, with an increase in the concentration, the kinetic constant decreased and the total removal amount increased. Therefore, it is speculated that the initial OTC treatment concentration is 40 mg/L, which is close to the limit of the O_3_/CaO_2_/${\mathrm{HCO}}_{3}^{-}$ system. As shown in Figure 1b,d,e and Figure 2b,d, good linear correlations between the ln k and different parameters were obtained. Therefore, the establishment of an O_3_/CaO_2_/${\mathrm{HCO}}_{3}^{-}$ system can degrade OTC effectively.

### 2.6. Active Substance Analysis

#### 2.6.1. Role of ·OH, ^1^O_2_, ${\cdot O}_{2}^{-}$, and ${\cdot \mathrm{CO}}_{3}^{-}$

**Figure 3:**
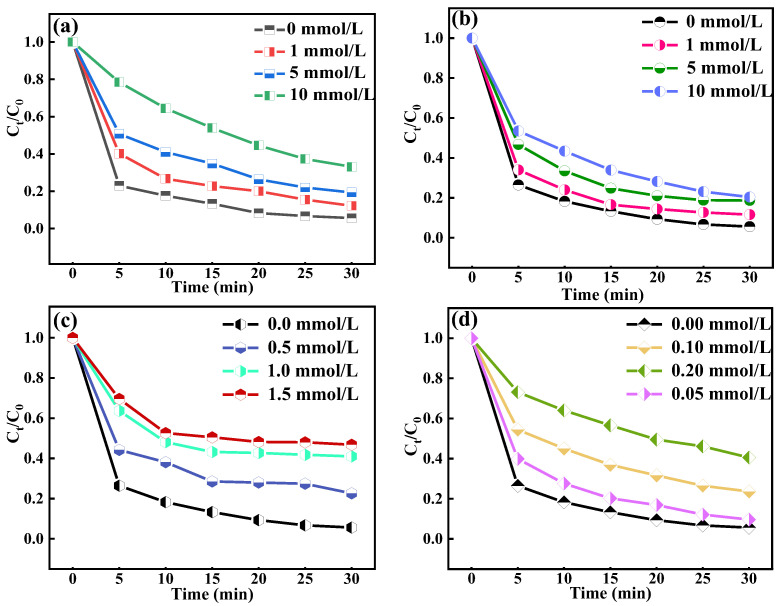
Effect of scavenger: (**a**) methanol; (**b**) DABCO; (**c**) P-benzoquinone; and (**d**) indole. Experimental conditions: [CaO_2_] = 0.05 g/L, [O_3_] = 0.75 g/h, [${\mathrm{HCO}}_{3}^{-}$] = 2.25 mmol/L, and [OTC] = 80 mmol/L.

Methanol is an efficient ·OH-trapping agent, which was used to capture ·OH in the O_3_/CaO_2_/${\mathrm{HCO}}_{3}^{-}$ system. As shown in Figure 3a, it can be seen that the degradation efficiency of OTC decreased with increasing amounts of methanol. When the methanol concentration changed from 0 mmol/L to 10 mmol/L, the degradation efficiency of OTC decreased from 94.0% to 66.9%. Meanwhile, the kinetic constant declined from 0.114 min^−1^ to 0.039 min^−1^ (Figure S1a). These results indicate that ·OH played an essential role in the degradation of OTC. ${\cdot O}_{2}^{-}$ can react to generate ^1^O_2_ (Equations (32)–(34)), which has a strong oxidation potential. 1,4-Diazabicyclooctane triethylenediamine (DABCO) is a highly efficient ^1^O_2_-trapping agent that can capture the ^1^O_2_ existing in the solution. Therefore, this section intends to use DABCO to study the role of ^1^O_2_ in the O_3_/CaO_2_/${\mathrm{HCO}}_{3}^{-}$ system.

(32) O2−+·OH → O12+OH−

(33) ·O2−+HO2+H+ → O12 + H2O2

(34) ·HO2+HO2 → O12+H2O2

Figure 3b shows the effect of various DABCO dosages on OTC elimination. Higher DABCO dosages will inhibit the degradation effect more obviously. When the addition of DABCO changed from 0 mmol/L to 10 mmol/L, the degradation efficiency of OTC decreased from 94.0% to 79.6%, with the kinetic constant varying from 0.114 min^−1^ to 0.060 min^−1^ (Figure S1b). DABCO has a significant inhibitory effect on OTC elimination. It is further inferred that ^1^O_2_ is actively involved in OTC degradation.

P-benzoquinone is a highly efficient ${\cdot O}_{2}^{-}$-trapping agent which can capture the ${\cdot O}_{2}^{-}$ present in a solution. After adding the catalyst CaO_2_ to the solution, CaO_2_ and H_2_O will produce a large quantity of ${\cdot O}_{2}^{-}$ (Equation (6)), which has a significant effect on OTC elimination. Figure 3c shows the effect of p-benzoquinone addition on OTC elimination. The degradation efficiency of OTC decreased with the p-benzoquinone addition. When the addition of p-benzoquinone was 0 mmol/L 0.5 mmol/L, 1.0 mmol/L, and 1.5 mmol/L, the degradation efficiency of OTC decreased from 94.0% to 53.2%, and the corresponding kinetic constant varied from 0.114 min^−1^ to 0.059 min^−1^, 0.039 min^−1^, and 0.033 min^−1^ (Figure S1c). This result showed that ${\cdot O}_{2}^{-}$ played a key role in the degradation of OTC.

Indole is an efficient ${\cdot \mathrm{CO}}_{3}^{-}$ capture agent, which can capture the ${\mathrm{HCO}}_{3}^{-}$ existing in a solution. After adding the catalyst NaHCO_3_ to the solution, NaHCO_3_ and H_2_O will produce part of ${\cdot \mathrm{CO}}_{3}^{-}$ (Equation (19)). When the indole concentration changed from 0 mmol/L to 0.2 mmol/L, the degradation efficiency of OTC decreased from 94.0% to 59.5% (Figure 3d), and the corresponding kinetic constant varied from 0.114 min^−1^ to 0.033 min^−1^ (Figure S1d). This result shows that ${\cdot \mathrm{CO}}_{3}^{-}$ is very involved in the degradation of OTC. The reaction rates of p-benzoquinone with ·OH and ·O_2_^−^ were 1.2 × 10^9^ and 8.3 × 10^8^ M^−1^ s^−1^, respectively, which are comparable. The reaction rate of indole with ·OH was 7.9 × 10^−14^ M^−1^s^−1^, which can be ignored. Because of their low reaction rates, P-benzoquinone and Indole can be regarded as not reacting with ·OH [48,49]. As shown in Figure S2, compared with different active substances, ·O_2_ made the greatest contribution to the degradation process of OTC, with 41.2% of the OTC being degraded by ·O_2_.

#### 2.6.2. Formation of Active Species

**Figure 4:**
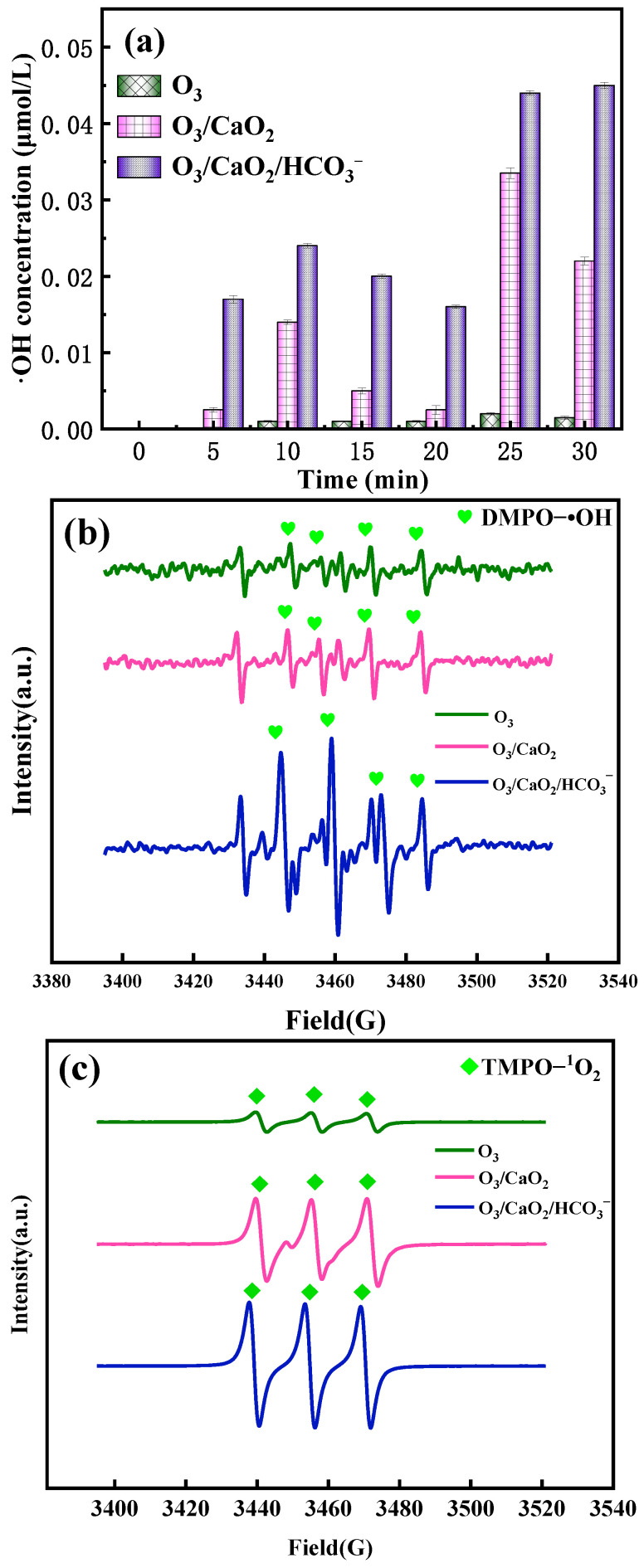
Formation of ·OH (**a**); ESR spectra of (**b**) ·OH and (**c**) ^1^O_2_.

To further explore the synergetic mechanism, the formation of ·OH under different systems was compared. The results are depicted in Figure 4a. Compared to the sole O_3_ system, the ·OH concentration was seriously increased when CaO_2_ was added. These phenomena verified that O_3_ could react with the H_2_O_2_ released by CaO_2_ and promote the formation of ·OH [50]. As desired, the ·OH concentration was further enhanced when ${\mathrm{HCO}}_{3}^{-}$ was added. The production of ·OH followed this law: O_3_/CaO_2_/NaHCO_3_ > O_3_/CaO_2_ > O_3_. Therefore, it can be explained that the higher degradation efficiency may have come from the higher production of ·OH. It is worth noting that the generation of ·OH in the O_3_/CaO_2_ system and the O_3_/CaO_2_/${\mathrm{HCO}}_{3}^{-}$ system showed a trend of first rising, then decreasing, and lastly increasing over time. It was speculated that the conversion of H_2_O_2_ into ·OH was violent, and a large amount of ·OH was generated within 5 min. ·OH could react with H_2_O_2_, and a certain amount of ·OH production was consumed from 5 to 20 min. After the two reactions reached equilibrium, ·OH production increased and tended to stabilize.

ESR analyses further revealed that there were obvious signals of the ·OH peak in various systems (Figure 4b). It can be seen that the characterized peaks of ·OH followed as: O_3_/CaO_2_/${\mathrm{HCO}}_{3}^{-}$ > O_3_/CaO_2_ > O_3_, which illustrated that ·OH was beneficial to generate in O_3_/CaO_2_/${\mathrm{HCO}}_{3}^{-}$ system. Figure 4c presents the characteristic peaks of ^1^O_2_ in various systems. It can be seen that ^1^O_2_ can be generated in O_3_, O_3_/CaO_2_, and O_3_/CaO_2_/${\mathrm{HCO}}_{3}^{-}$ systems. Compared to sole O_3_, the characteristic peaks of ^1^O_2_ were enhanced with the addition of CaO_2_ and ${\mathrm{HCO}}_{3}^{-}$, which indicates that the addition of CaO_2_ and ${\mathrm{HCO}}_{3}^{-}$ effectively increased the production of ^1^O_2_.

### 2.7. OTC Degradation Process

#### 2.7.1. UV-Vis Spectra

Figure S3 shows the UV-Vis spectrum results of the OTC solution in the sole O_3_ system and O_3_/CaO_2_/${\mathrm{HCO}}_{3}^{-}$ system. The OTC spectrum shows two absorption bands near 276 nm and 353 nm, and the absorption peak near 353 nm is the characteristic peak of OTC. The common point of the two systems is that, after 30 min of processing, the characteristic peaks of OTC were significantly reduced, and the OTC molecules were weakened. The π–π * transition of its heteroaromatic ring makes the main contribution to this [51,52]. Compared with Figure S3a, the characteristic peak of OTC in Figure S3b has a more rapid decline, which indicates that O_3_/CaO_2_/${\mathrm{HCO}}_{3}^{-}$ had a better OTC degradation ability.

#### 2.7.2. 3D EEMFS

**Figure 5:**
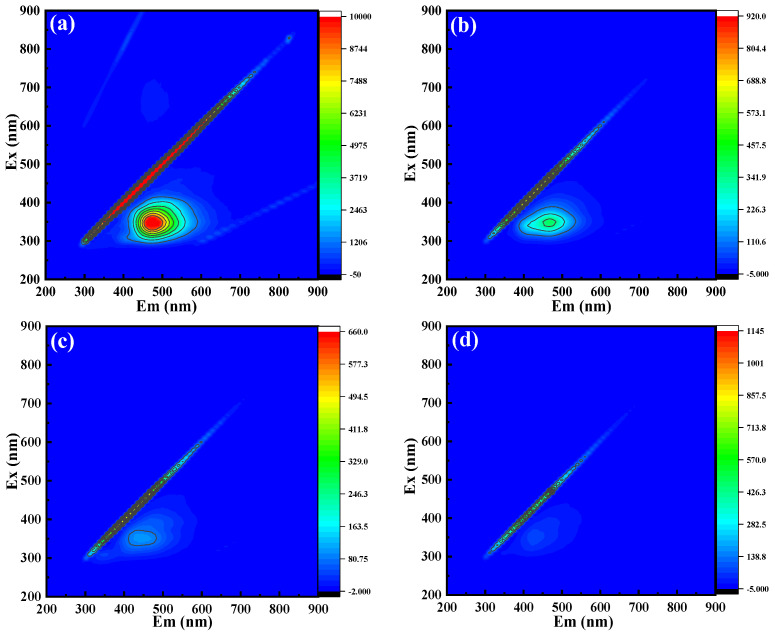
3D EEMF spectra of OTC samples under different treatment times: (**a**) 0 min; (**b**) 10 min; (**c**) 20 min; abd (**d**) 30 min.

To study the degradation process of OTC more deeply, 3D EEMFs were used for characterization. The emission wavelength and the excitation wavelength were all set as 200–600 nm, whose sampling intervals were 5 nm and 10 nm, respectively. Figure 5a is taken from the original OTC sample, and Figure 5b,c are OTC samples processed for 10 min, 20 min, and 30 min, respectively. In the original OTC solution, there was a prominent fluorescence peak at E_x_/E_m_ = 300–400/400–600 nm, which is located in the humic acid-like fluorescence region [53]. With the passage of time, the characteristic peak intensity gradually weakened and the fluorescence intensity dropped from 2000.23 to 220.18, indicating that the OTC molecule was destroyed and may have been mineralized into some intermediates or decomposed into CO_2_ and H_2_O (Figure 5d). The 3D EEMFs results show that the O_3_/CaO_2_/${\mathrm{HCO}}_{3}^{-}$ system can degrade OTC, which is consistent with the UV-Vis spectrum results.

#### 2.7.3. Variation of TOC and COD

**Figure 6:**
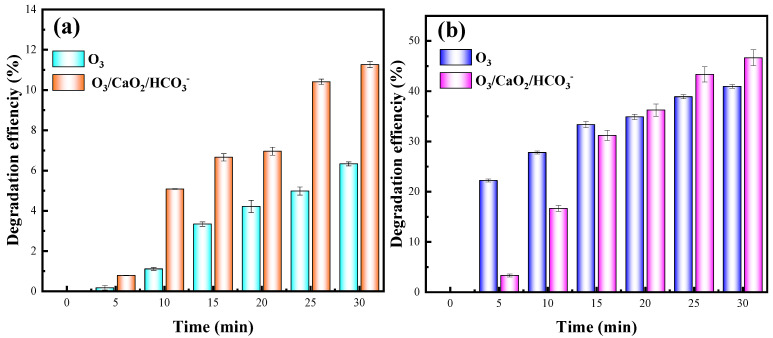
Degradation efficiency of (**a**) TOC and (**b**) COD during OTC degradation.

TOC is an important parameter for evaluating the mineralization ability of oxidation systems. Figure 6a shows the degradation of OTC in terms of TOC removal, with or without two catalysts in the O_3_ oxidation system. The carbon-containing organic matter in the OTC solution decreased as the treatment time increased. After the two catalysts were added, the TOC concentration decreased. When the treatment time was 30 min, the TOC degradation efficiency could reach 14.7%. Figure 6b depicts the COD concentration in the two systems. The COD concentration of the sole O_3_ system underwent a slight drop, the speed was plodding, and it tended to be stable. The O_3_/CaO_2_/${\mathrm{HCO}}_{3}^{-}$ system had a very high COD concentration at 0 min, with a significant drop and a fast speed, and a COD degradation efficiency of 48.8% was obtained within 30 min of the processing time. This O_3_/CaO_2_/${\mathrm{HCO}}_{3}^{-}$ system produced a large number of active free radicals, which actively participated in the degradation process, which supports the derivation of the mechanism in the previous chapter. The O_3_/CaO_2_/${\mathrm{HCO}}_{3}^{-}$ system has an excellent OTC degradation ability, but the degradation efficiency of TOC is far below 100%. Combining the results of the UV-Vis spectroscopy and 3D EEMFs, we speculated that a great deal of intermediates were formed during OTC degradation.

#### 2.7.4. Variation of pH and Conductivity

Figure S4a shows the pH change over time during OTC degradation. The O_3_/CaO_2_/${\mathrm{HCO}}_{3}^{-}$ system presented a slightly alkaline environment, while the sole O_3_ system presented a neutral environment. As the treatment time increased, the pH values in both systems showed a gentle downward trend. Figure S4b shows the change in conductivity during OTC degradation. The conductivity of the sole O_3_ system was 21.2 μS/cm at 0 min, and after 30 min of treatment, the conductivity increased slightly to 30.3 μS/cm. The conductivity of the O_3_/CaO_2_/${\mathrm{HCO}}_{3}^{-}$ system was 308 μS/cm at 0 min and dropped to 262 μS/cm after 30 min of treatment. We speculate that the addition of catalysts produced more inorganic ions, and acidic free radicals were generated during the degradation of OTC. In the process of degradation, ${\mathrm{HCO}}_{3}^{-}$ reacted with H_2_O_2_ to form ${\mathrm{HCO}}_{4}^{-}$ on the one hand and ·OH to form ${\cdot \mathrm{CO}}_{3}^{-}$ on the other, so ${\mathrm{HCO}}_{3}^{-}$ itself was consumed massively. The decrease in conductivity may have been due to the consumption of ${\mathrm{HCO}}_{3}^{-}$.

### 2.8. DFT Analysis and Degradation Pathway

**Figure 7:**
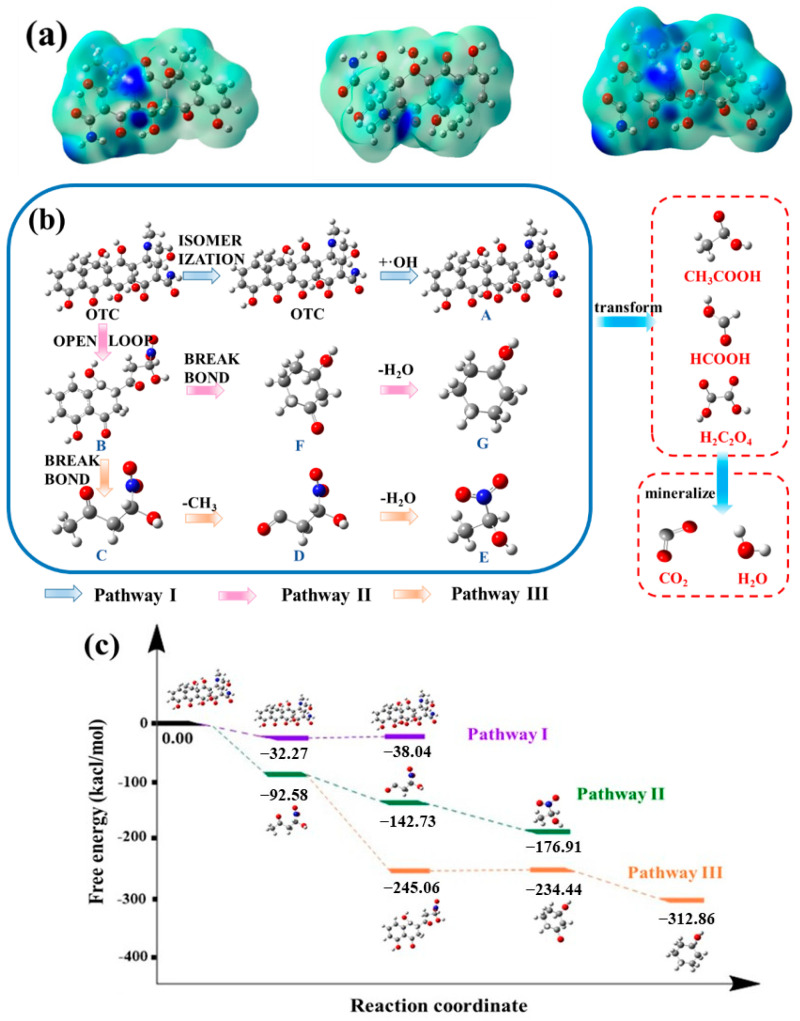
(**a**) Fukui function of OTC mapped electron density isosurface: f^−^; f^+^; and f^0^, (**b**) pathways for OTC degradation and (**c**) energy barrier diagram. (Gray atoms represent C, white atoms represent H, red atoms represent N, and blue atoms represent O).

The Fukui functions calculated with density functional and electron orbitals provide good evidence for predicting reaction sites. The molecular structure of OTC was calculated with the Gaussian 09 program. The Fukui function of OTC mapped the electron density isosurface and the calculation results are provided in Figure 7a and Table S2. Now, it is generally accepted that, the higher the f^+^ function of the atom is, the easier will it be nucleophilically attacked; the higher the f^−^ function of the atom is, the easier will it be electrophilically attacked; and the higher the f^0^ function of the atom is, the easier will it be radically attacked. To indicate the possibility of the site being attacked clearly based on the above mechanism, electron cloud diagrams were drawn. It can be seen that the highlight is above C21 and O26, which means that these two sites would be easy to electrophilically attack. For the same reason, C4, O12, and C20 were easier to nucleophilically attack. C21 and O26 were easier to radically attack. Through a comprehensive comparison of the Fukui function table, it is inferred that OTC molecules may be decomposed into A (pathway 1) and B (pathway 2) due to electron and free radical attacks on C21 and O26.

To deeply explore the OTC degradation process in the O_3_/CaO_2_/${\mathrm{HCO}}_{3}^{-}$ system, a Liquid Chromatograph Mass Spectrometer (LC-MS) (ES+, ES−) was adopted and the results are illustrated in Figure S5. According to the results of LC-MS, it can be inferred that there were eight main intermediates: A C_22_H_24_N_2_O_10_ (*m*/*z* = 477), B C_15_H_13_NO_7_ (*m*/*z* = 296), C C_4_H_7_NO_4_ (*m*/*z* = 132), D C_3_H_7_NO_4_ (*m*/*z* = 118), E C_3_H_7_NO_3_ (*m*/*z* = 106), F C_6_H_10_O_2_ (*m*/*z* = 113), and G C_6_H_12_O (*m*/*z* = 101). The molecular structures of the eight intermediates are shown in Table S3. Combined with the Density Functional Theory (DFT) calculation and LC-MS detection results, the inferred degradation path of OTC is shown in Figure 7b. Figure 7c shows a free energy ladder diagram of these reactions. The initial energy of OTC was 0 kcal/mol, and the major degradation pathways are represented by negative energy levels. These reactions may occur, in theory, by different radicals. In the OTC molecular structure formula, weak chemical bonds between atoms are easy to destroy. C21 in pathway I is prone to enol ketone isomerization, and OTC is transformed into more stable isomers. The ketone/enol portion of C21–C25 is vulnerable to ·OH attack, and further hydroxylation by-product A is obtained. O_3_ in the system is an electrophilic reagent, which tends to attack the electron-rich region in the OTC structure [54]. The double-bond structure of C21–C25 in pathway II and pathway III is broken by free radicals such as O_3_, and the main product is B. Fragile C5a breaks, causing B molecules to decompose into C and F. F is further dehydrated to obtain by-product G. C is demethylated to obtain by-product D, and D is further dehydrated to obtain small molecule by-product E. These intermediates could be further oxidized to small molecular organic acids, and ultimately mineralized to CO_2_ and H_2_O [55,56].

### 2.9. Toxicity Evaluation

**Figure 8:**
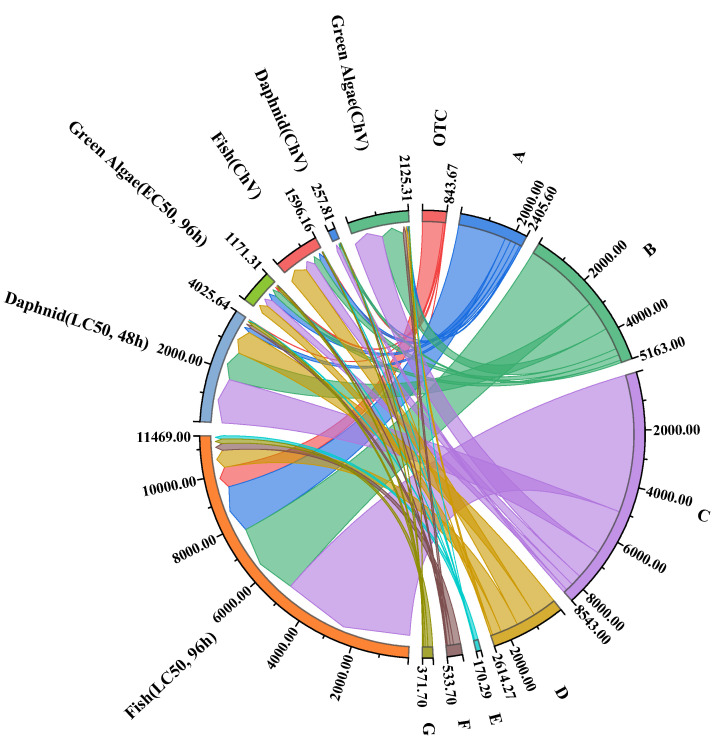
Toxicity of OTC degradation intermediates. (A: C_22_H_24_N_2_O_10_; B: C_15_H_13_NO_7_; C: C_4_H_7_NO_4_; D: C_3_H_7_NO_4_; E: C_3_H_7_NO_3_; F: C_6_H_10_O_2_; G: C_6_H_12_O).

The U.S. Environmental Protection Agency’s toxicity evaluation software tool (US–EPA–TEST, version 5.1.2) program of this study predicts the acute and chronic toxicity of OTC and its intermediates [55,57]. The European Union criteria standard is adopted for acute toxicity, and the Chinese guidelines for the evaluation of hazardous chemicals (HJ/TI 154—2004) are adopted for chronic toxicity. The test results are shown in Table S4 and Figure 8. The daphnid LC50 of OTC is 9.34 mg/L, belonging to “toxic”. After 30 min of treatment in this system, the toxicity of intermediates was significantly reduced, and all intermediates except F were located in “harmless” or “harmful” areas. A, B, and C were located in the “Harmless” area. Therefore, it can be speculated that the toxicity of OTC will decline significantly after O_3_/CaO_2_/${\mathrm{HCO}}_{3}^{-}$ treatment, and detoxification may be achieved.

### 2.10. Feasible for Treatment of Other Antibiotics and Actual Wastewater

**Figure 9:**
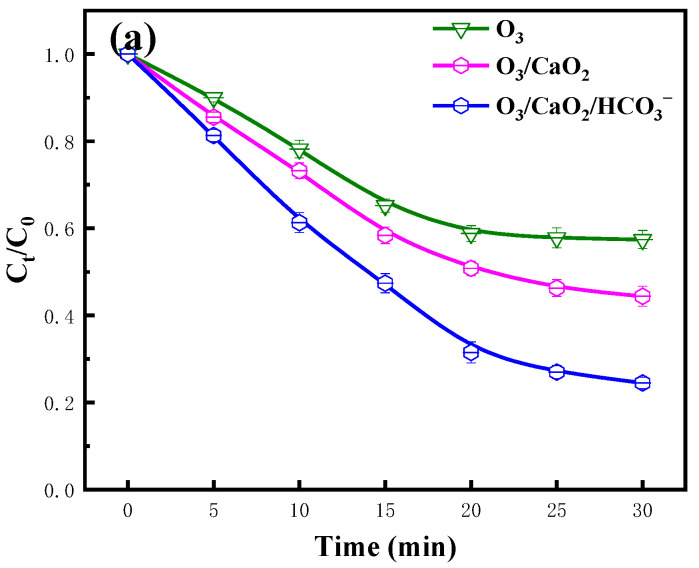

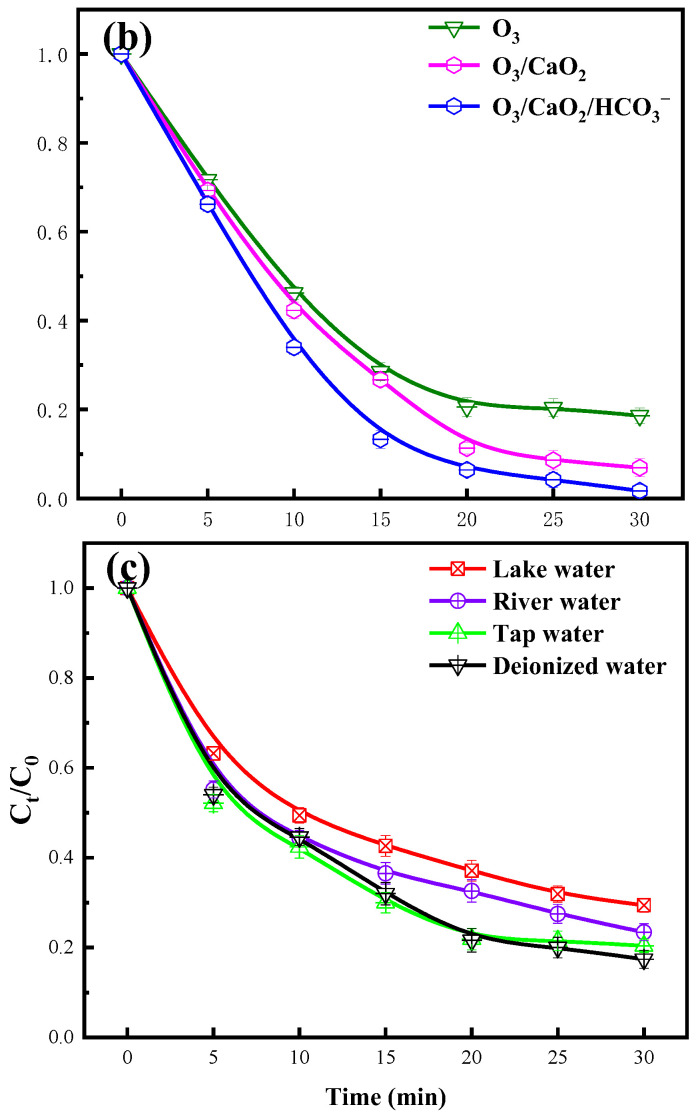
Degradation of (**a**) BPA and (**b**) TC; (**c**) actual wastewater treatment situation.

The feasibility of O_3_/CaO_2_/${\mathrm{HCO}}_{3}^{-}$ for the treatment of other antibiotics and actual wastewater was investigated. The degradation of bisphenol A (BPA) and tetracycline (TC) under different systems is presented in Figure 9a,b. No matter the aspect of BPA or TC, the highest degradation efficiency was obtained in the O_3_/CaO_2_/${\mathrm{HCO}}_{3}^{-}$ system. As a whole, it can be seen that the degradation efficiency could reach above 80%, illustrating that the O_3_/CaO_2_/${\mathrm{HCO}}_{3}^{-}$ system is also adapted to other antibiotic degradations. Figure 9c depicts the degradation of OTC in various water bodies. Over time, OTC molecules are gradually degraded in different water bodies such as deionized water, lake water, river water, and tap water. The degradation effect has not undergone significant changes, indicating that the system is also suitable for the treatment of actual wastewater.

### 2.11. Energy Efficiency Evaluation

Energy efficiency assessment is a part that cannot be underestimated. It is closely related to whether a new system can be promoted and applied. Therefore, we calculated the energy efficiency of the sole O_3_ system, O_3_/CaO_2_ system, and O_3_/CaO_2_/${\mathrm{HCO}}_{3}^{-}$ system in different program doses, and the results are shown in Table S5. In the sole O_3_ system, the energy efficiency reached 1.55 g/kWh. When 0.050 g/L of CaO_2_ was added, the energy efficiency increased to 1.82 g/kWh. On this basis, when adding 2.25 mmol/L of ${\mathrm{HCO}}_{3}^{-}$, the optimal energy efficiency could be increased to 1.91 g/kWh. The energy efficiency of the optimal O_3_/CaO_2_/${\mathrm{HCO}}_{3}^{-}$ system could reach 1.91 g/kWh, which is an impressive result.

Table S6 lists the comparison results of the kinetic constants and energy efficiencies of other degradation OTC processes and this process. The degradation efficiency of the Al_0_-Gr-Fe_0_/O_2_ Fenton process for OTC was higher than that of other systems, close to 100%, but the energy efficiency was extremely low [40]. CO_3_O_4_/CNT catalyzed the degradation of OTC, and its degradation efficiency was close to O_3_/CaO_2_/${\mathrm{HCO}}_{3}^{-}$, but required a longer processing time and a relatively small kinetic constant [43]. Among the many degradation OTC processes, O_3_/CaO_2_/${\mathrm{HCO}}_{3}^{-}$ is undoubtedly the most economical and has the most potential [41,42,44,45].

## 3. Materials and Methods

### 3.1. Chemicals

OTC, CaO_2_, NaOH, triethylenediamine, terephthalic acid, sodium indigo disulfonate, BPA, and TMPO were all analytical grade (AR), and were bought from Aladdin Reagent (Aladdin Reagent (Shanghai) Co., Ltd., Shanghai, China). H_2_SO_4_ (98%) and H_3_PO_4_ were purchased from Sinopharm Chemical Reagent (Sinopharmaceutical Group Chemical Reagent (Shanghai) Co., Ltd., Shanghai, China). Indole, TC, and DMPO were obtained from Macklin (McLean biochemical Technology (Shanghai) Co., Ltd., Shanghai, China). NaHCO_3_ of analytical grade (AR). was obtained from Lingfeng Chemical Reagent (Lingfeng Chemical Reagent (Shanghai) Co., Ltd., Shanghai, China). None of the chemicals required further purification. All of the solution was made with deionized water produced by an ultra-pure water system (Biosafer, Biosafer—10R, Saifei (Nanjing) Co., Ltd., Nanjing, China).

### 3.2. Experimental Process

A reactor schematic diagram is presented in Figure S6. The O_3_ was generated by plasma discharge, which was named as plasma-O_3_ generator (CQ, Changqing—802S). The amount of O_3_ used was extracted and adjusted using an atmospheric sampler. The active species were characterized using an electron paramagnetic resonance spectrometer (BRUKER, MESRV30077, Brooke Technology (Beijing) Co., Ltd., Beijing, China). To monitor the OTC degradation process, 3 mL of the aqueous solution was taken out of the reaction mixture every 5 min, and the experiment was repeated several times to take the average value for the subsequent analysis. The best degradation conditions were as follows: the concentration of CaO_2_ was 0.050 g/L, the dosage of O_3_ was 0.75 g/h, the dosage of ${\mathrm{HCO}}_{3}^{-}$ was 2.25 mmol/L, the pH was 2.4, and the initial concentration of OTC was 40 mg/L.

### 3.3. Analysis

The OTC calculation and analysis process is provided in Text S1. For the detection of O_3_, ·OH, and H_2_O_2_ in the OTC degradation process, the indigo method [58], the terephthalic acid probe method [59], and the titanyl sulfate method [60] were used, respectively. The data were analyzed and plotted with the origin (OriginLab Inc., version 9.8.0.200, Northampton, MA, USA) software.

### 3.4. DFT Analysis and Toxicity Evaluation

The molecular structures of the organic compounds studied were built using the Gauss View 6.0 software (Gaussian Inc., Wallingford, CT, USA). The built molecular structures were then optimized using chemical DFT methods (B3LYP/6-311++G (d, p)), employing Gaussian 16 (Gaussian Inc., Wallingford, CT, USA). Detailed information is shown in Text S2. US-EPA-TEST (US EPA, version 5.1.2, USA) was adopted to assess the toxicity of OTC and its intermediates based on the bioaccumulation factor and LC50 [57].

## 4. Conclusions

This study constructed O_3_/CaO_2_/${\mathrm{HCO}}_{3}^{-}$ oxidation technology to degrade OTC wastewater. The introduction of CaO_2_/${\mathrm{HCO}}_{3}^{-}$ significantly promoted the degradation of OTC in the O_3_ process in terms of degradation efficiency and kinetic constants. However, an excessive CaO_2_/${\mathrm{HCO}}_{3}^{-}$ dosage had an inhibitory effect on OTC degradation. The greater the O_3_ flow rate, the lower the OTC concentration, and the higher the pH, which were beneficial for OTC decomposition. Active substances such as ·OH, ${\cdot O}_{2}^{-}$, ^1^O_2_, and ${\cdot \mathrm{CO}}_{3}^{-}$ participated in the degradation of OTC. The enhanced degradation of OTC was mainly due to the high production of ·OH. After the treatment of the O_3_/CaO_2_/${\mathrm{HCO}}_{3}^{-}$ system, TOC and COD had both declined. However, as the reaction time increased, the pH value was reduced, with or without catalysts. The conductivity was enhanced without a catalyst, while an opposite trend was observed for the O_3_/CaO_2_/${\mathrm{HCO}}_{3}^{-}$ system. According to a DFT analysis, LC-MS, and related studies, the degradation pathways related to OTC degradation were proposed. After the O_3_/CaO_2_/${\mathrm{HCO}}_{3}^{-}$ system treatment, the toxicity of OTC could be reduced. O_3_/CaO_2_/${\mathrm{HCO}}_{3}^{-}$ is also feasible for the treatment of other antibiotics and actual wastewater. The energy efficiencies for OTC degradation under various systems were compared. This research showed that the O_3_/CaO_2_/${\mathrm{HCO}}_{3}^{-}$ system could be considered as an efficient and commercial oxidation process for OTC treatment.

## Data Availability

Data are contained within the article and Supplementary Materials.

## Supplementary Materials

The following supporting information can be downloaded at: https://www.mdpi.com/article/10.3390/molecules29030659/s1. Figure S1. First-order kinetic fitting and kinetic constants of scavenger: (a) methanol; (b) DABCO; (c) p-benzoquinone; and (d) indole. Figure S2. Contribution rates of different free radicals. Figure S3. UV-Vis curve of OTC degradation in (a) O_3_ system and (b) O_3_/CaO_2_/${\mathrm{HCO}}_{3}^{-}$ system. Figure S4. Variation in (a) pH and (b) conductivity during OTC degradation. Figure S5. MS spectra of OTC degradation intermediates identified by LC-MS. Figure S6. Schematic representation of the O_3_ reactor for the oxidative degradation of OTC. Table S1. Oxidation potential of active radical species in O_3_/CaO_2_/${\mathrm{HCO}}_{3}^{-}$ system. Table S2. Fukui function of OTC. Table S3. The proposed structure information of OTC degradation products. Table S4. Toxicity of OTC degradation intermediates. Table S5. Energy efficiency during OTC elimination with various CaO_2_ and NaHCO_3_ dosages. Table S6. Comparison with other techniques on OTC elimination. References [40,41,42,43,44,45] are cited in the supplementary materials.
