# Supplementary material for: The Degradation of Aqueous Oxytetracycline by an O3/CaO2 System in the Presence of HCO3−: Performance, Mechanism, Degradation Pathways, and Toxicity Evaluation
Source: Molecules. 2024 Jan 31;29(3):659. doi: 10.3390/molecules29030659 (PMC10856086; doi:10.3390/molecules29030659)
Supplement: Supplementary file 1 [file molecules-29-00659-s001.zip › molecules-2819980-supplementary.pdf]

Supplementary Material

The Degradation of Aqueous Oxytetracycline by an O_3/CaO_2 System in the Presence of HCO_3^-: Performance, Mechanism, Degradation Pathways, and Toxicity Evaluation

Zedian Li [1,†], Liangrui Xiang [2,†], Shijia Pan [2], Dahai Zhu [1], Shen Li [3] and He Guo [2,*]

[1] School of Energy and Materials, Shanghai Polytechnic University, Shanghai 201209, China; lzd111303@outlook.com (Z.L.); zhudh@sspu.edu.cn (D.Z.)
[2] College of Biology and the Environment, Nanjing Forestry University, Nanjing 210037, China; 17755053066@163.com (L.X.); 13675128810@163.com (S.P.)
[3] Anhui Jiuwu Tianhong Environmental Protection Technology Co., Ltd., Hefei 230011, China; lishen3569@outlook.com
* Correspondence: heguo@njfu.edu.cn
† These authors contributed equally to this work.

Text S1 OTC calculation and analysis process

The prepared OTC solution was analyzed using an ultraviolet visible spectrophotometer (METASH，UV-5500PC) to monitor the attenuation of its characteristic absorbance at 353 nm. The OTC degradation kinetics were in accordance with the first-order kinetic model, and the relationship between the degradation kinetics and OTC concentration was as follows:

$$Ln(C_0/C_t) = kt \tag{S1}$$

In the formula, t is the reaction time, min; C_0 is the concentration of OTC in 0 min, mg/L; C_t is the concentration of OTC at time t, mg/L; and k is the kinetic constant, min^{-1}.

The degree of mineralization of OTC degradation was evaluated with a total organic carbon detector (SHMADZU, TOC-V). A water quality detector (Lianhua, 5B-6C) was adopted to determine the chemical oxygen demand (COD). Changes in conductivity and pH during the degradation process were measured with a conductivity meter (INESA, DDS-307A) and acidity meter (INESA, PHS-3C), respectively. The 3D (three-dimensional) EEMF (excitation emission matrix fluorescence spectrum) of the OTC solution was marked by a fluorescence spectrometer (Hitachi, FL4500), and the UV-Vis spectrum was marked by a UV-Vis spectrophotometer (Lianhua, UV-5500PC).

Text S2 DFT analysis

The molecular structure of OTC was calculated with the Gaussian 09 program. To obtain the Fukui Function by npa charge, the B3LYP/6-31G(d) pop = npa statement was used. The Multiwfn and Gaussian view programs were used to gain the final electron cloud diagrams. In order to confirm the degradation intermediates and predict the possible degradation pathway, a DFT analysis was carried out by the means of the

Fukui Function.

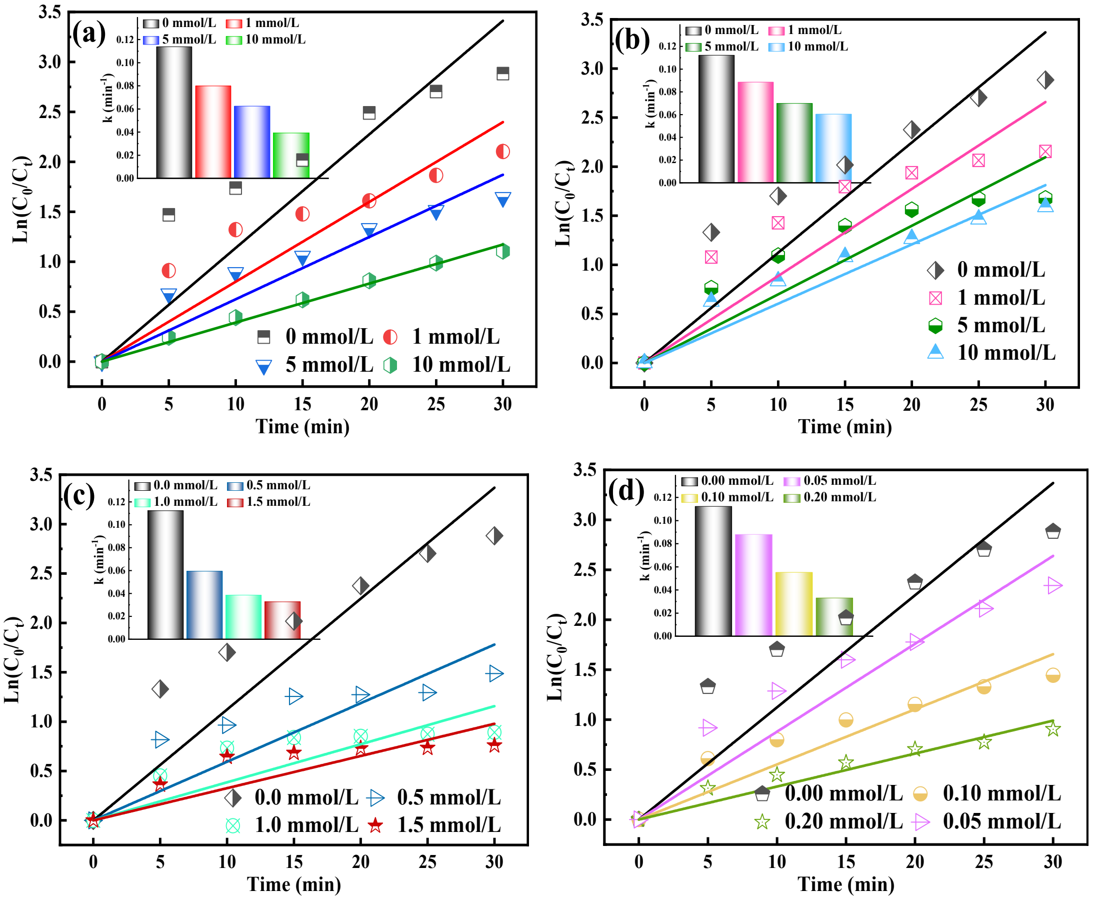

Figure S1 First-order kinetic fitting and kinetic constants of scavenger: (a) methanol; (b) DABCO; (c) p-benzoquinone; and (d) indole.

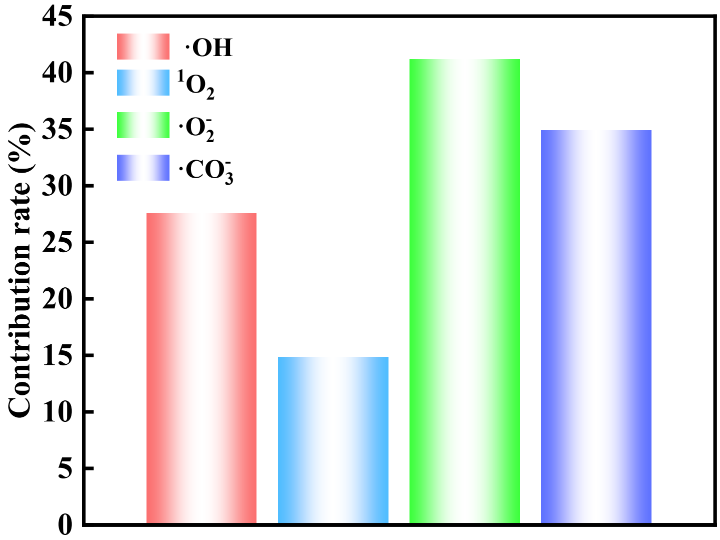

Figure S2 Contribution rates of different free radicals.

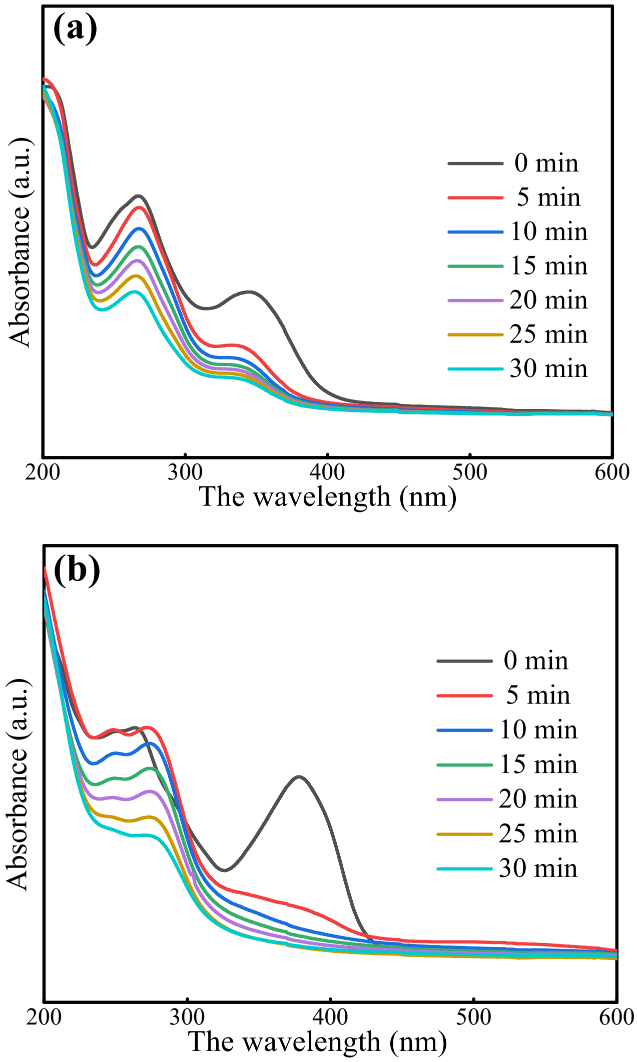

Figure S3 UV-Vis curve of OTC degradation in (a) O_3 system and (b) $O_3/CaO_2/HCO_3^-$ system.

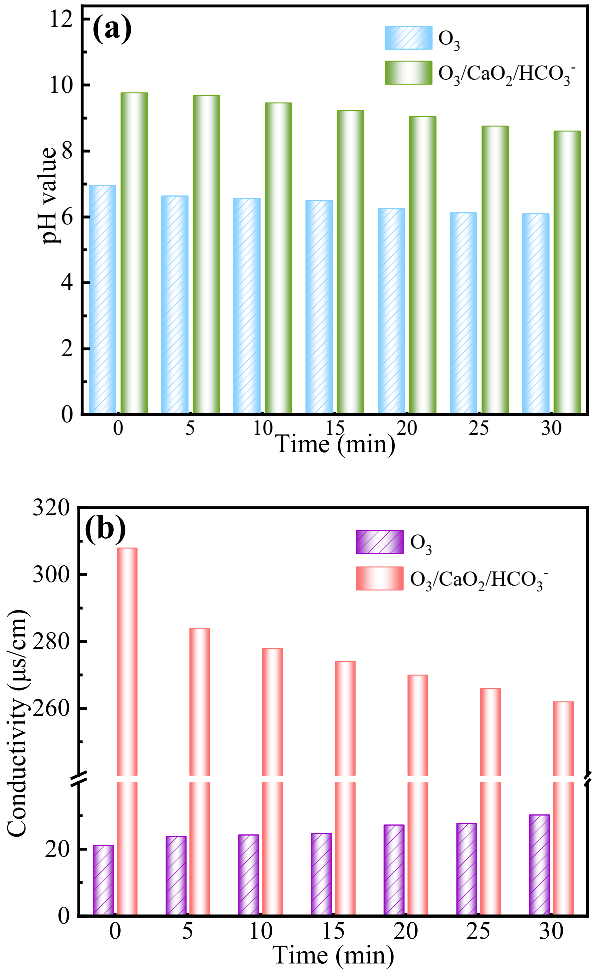

Figure S4 Variation in (a) pH and (b) conductivity during OTC degradation.

MS ES+: m/z=105

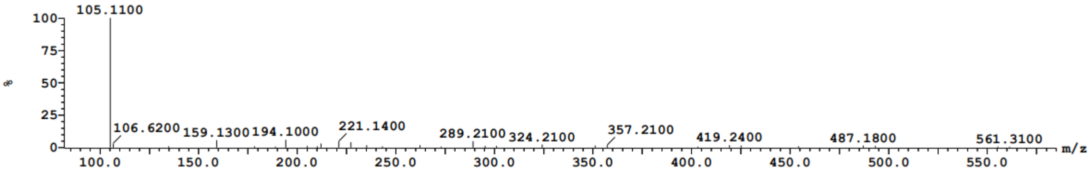

MS ES+: m/z=130

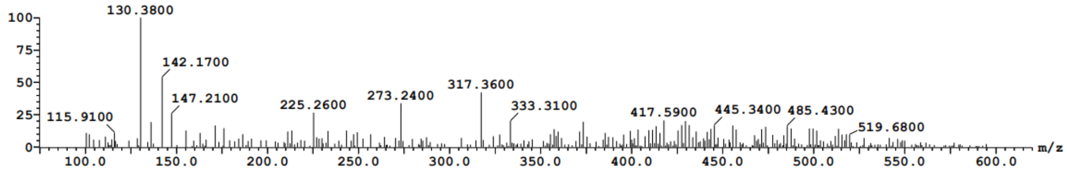

MS ES+: m/z=105

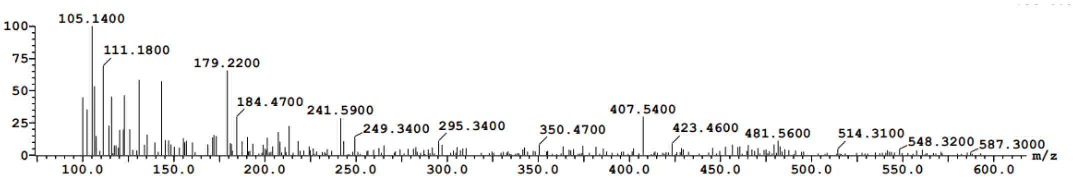

MS ES+: m/z=101

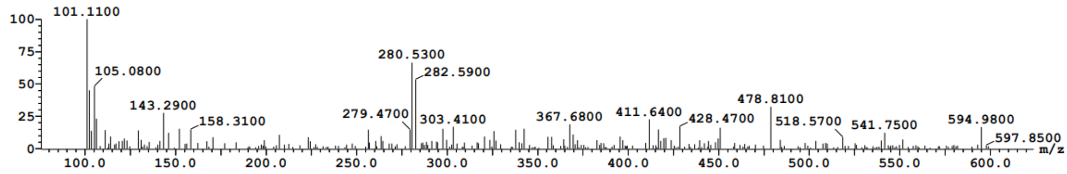

MS ES+: m/z=477

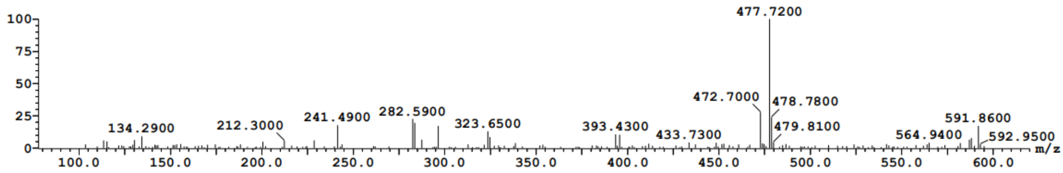

MS ES+: m/z=297

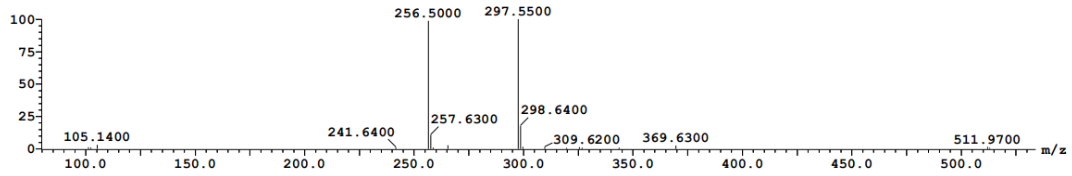

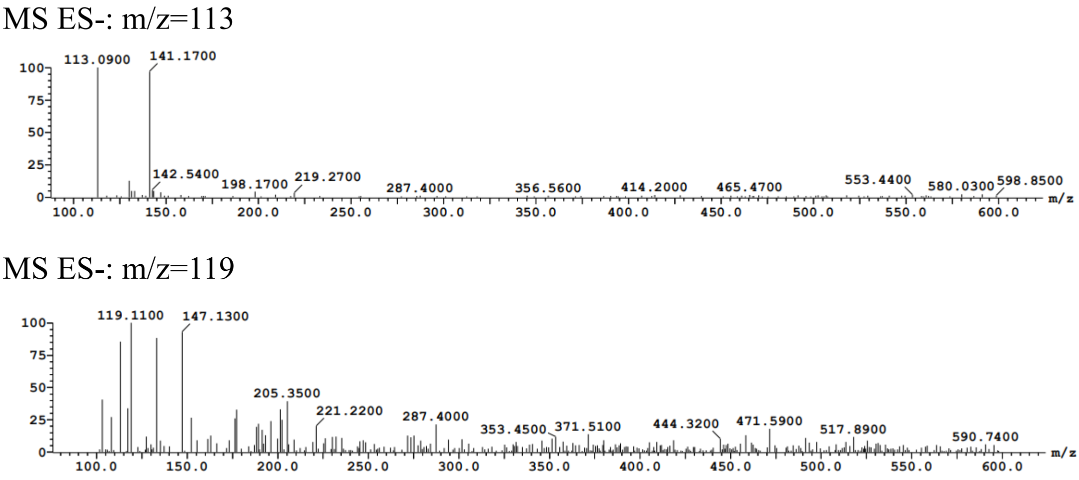

Figure S5 MS spectra of OTC degradation intermediates identified by LC-MS.

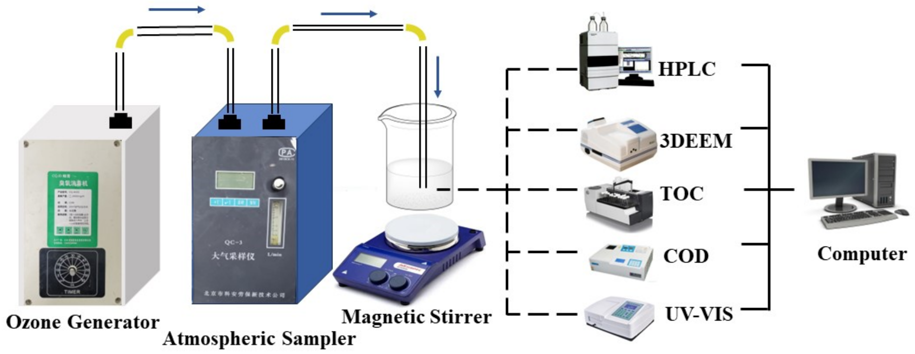

Figure S6 Schematic representation of the O_3 reactor for the oxidative degradation of OTC.

Table S1 Oxidation potential of active radical species in $O_3/CaO_2/HCO_3^-$ system.

Radical	Oxidation potential (V)
$\cdot OH$	2.80
$\cdot H$	1.66
$\cdot O_2^-$	0.94
$\cdot O_3^-$	4.00
$^1O_2$	0.65
O_2	0.40
O_3	2.07
H_2O_2	1.76
$\cdot CO_3^-$	1.69

Table S2 Fukui function of OTC.

atom	f^-	f^+	f^0	atom	f^-	f^+	f^0
C1	0.0075	0.0168	0.0121	H30	0.0287	0.0173	0.0230
C2	-0.0095	0.0278	0.0092	N31	0.0007	0.0095	0.0051
C3	0.0044	-0.0001	0.0022	C32	-0.0046	-0.0021	-0.0033
C4	0.0155	0.0567	0.0361	H33	0.0323	0.0180	0.0251
C5	0.0116	0.0268	0.0192	H34	-0.0185	-0.0055	-0.0120
H6	0.0070	0.0172	0.0121	H35	0.0015	0.0076	0.0045
H7	0.0131	0.0251	0.0191	C36	-0.0053	-0.0020	-0.0036
H8	0.0134	0.0242	0.0188	H37	0.0025	0.0079	0.0052
O9	0.0023	0.0093	0.0058	H38	0.0118	0.0081	0.0100
H10	0.0101	0.0173	0.0137	H39	0.0199	0.0154	0.0177
C11	-0.0001	0.0027	0.0014	C40	-0.0031	0.0163	0.0066
O12	0.0127	0.0696	0.0412	O41	0.0380	0.0289	0.0334
C13	-0.0028	-0.0041	-0.0034	N42	0.0183	0.0220	0.0202
H14	0.0083	0.0118	0.0100	H43	-0.0054	0.0039	-0.0007
H15	0.0151	0.0188	0.0170	H44	0.0275	0.0194	0.0234
H16	-0.0017	0.0032	0.0008	O45	0.0552	0.0238	0.0395
C17	-0.0042	-0.0075	-0.0059	H46	0.0094	0.0074	0.0084
C18	-0.0296	-0.0030	-0.0163	C47	-0.0047	-0.0161	-0.0104
C19	-0.0117	0.0079	-0.0019	C48	-0.0137	0.1629	0.0746
C20	-0.0034	0.0638	0.0302	H49	0.0152	0.0248	0.0200
C21	0.3593	-0.0129	0.1732	O50	0.0404	0.0877	0.0641
C22	-0.0439	-0.0109	-0.0274	H51	0.0080	0.0095	0.0087
O23	0.0128	0.0039	0.0083	H52	0.0043	0.0156	0.0099
H24	0.0285	0.0071	0.0178	H53	0.0530	0.0133	0.0332
C25	0.0187	0.0114	0.0150	O54	-0.0001	0.0069	0.0035
O26	0.1910	0.0293	0.1102	H55	0.0031	0.0097	0.0064
C27	-0.0164	0.0275	0.0055	C30	0.0172	0.0314	0.0243
C28	0.0519	0.0286	0.0403	C31	0.0175	0.0322	0.0248
C29	-0.0114	0.0045	-0.0034	\			

Table S3 The proposed structure information of OTC degradation products.

Products	Formula	m/z	Molecular structures
OTC	$C_{22}H_{24}N_2O_9$	461	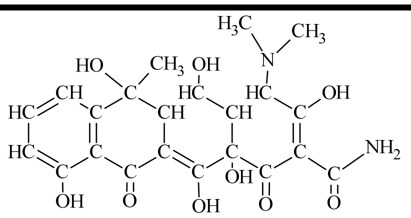
A	$C_{22}H_{24}N_2O_{10}$	477 (MS ES +)	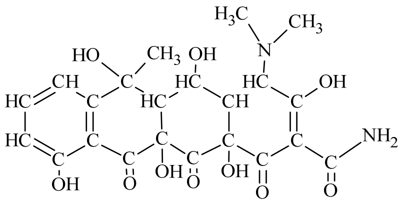
B	$C_{15}H_{13}NO_7$	297 (MS ES +)	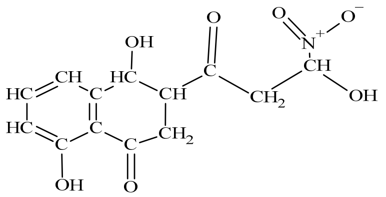
C	$C_4H_7NO_4$	130 (MS ES +)	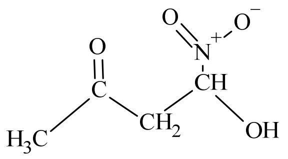
D	$C_3H_7NO_4$	119 (MS ES -)	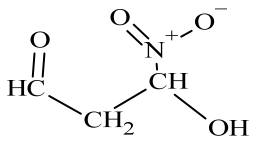
E	$C_3H_7NO_3$	105 (MS ES +)	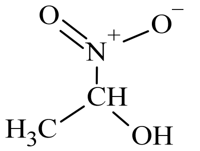
F	$C_6H_{10}O_2$	113 (MS ES -)	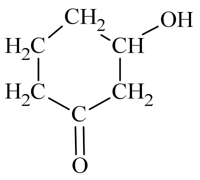
G	$C_6H_{12}O$	101 (MS ES +)	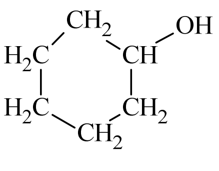

Table S4 Toxicity of OTC degradation intermediates.

Number	Acute toxicity (mg/L)[1]			Chronic toxicity (ChV, mg/L)[2]			ECOSAR class
	Fish (LC50, 96h)	Daphnid (LC50, 48h)	Green Algae (EC50, 96h)	Fish (ChV)	Daphnid (ChV)	Green Algae (ChV)	
OTC	757	9.34	63.7	3.36	2.36	7.91	Phenol Amines
A	1750	166	215	203	11.0	60.6	Aliphatic Amines
B	2760	1070	200	240	91.0	802	Ketone Alcohols
C	5000	1670	279	397	127	1070	Ketone Alcohols
D	646	871	325	701	4.47	66.8	Aldehydes (Mono)
E	126	27.6	2.41	10.9	2.48	0.896	Nitro Alcohols
F	256	116	24.2	24.5	11.0	102	Ketone Alcohols
G	174	95.7	62.0	16.4	8.50	15.1	Neutral Organics

Toxic **Harmful** **Harmless**

1, the European Union criteria, harmless (> 100mg/L), harmful (10-100 mg/L), and toxic (1-10 mg/L);

2, the Chinese hazard chemical evaluation guidelines (HJ/TI 154-2004), i.e., harmless (> 10 mg/L), harmful (1-10mg/L), and toxic (0.1-1 mg/L).

Table S5 Energy efficiency during OTC elimination with various CaO_2 and $NaHCO_3$ dosages.

Treatment system	CaO_2 dosage (g/L)	Energy efficiency (g/kWh)	Treatment system	$NaHCO_3$ dosage (mmol/L)	Energy efficiency (g/kWh)
O_3	——	1.55	O_3	——	1.55
O_3/CaO_2	0.025	1.72	O_3/CaO_2	0	1.82
O_3/CaO_2	0.050	1.82	$O_3/CaO_2/HCO_3^-$	0.89	1.87
O_3/CaO_2	0.063	1.73	$O_3/CaO_2/HCO_3^-$	2.25	1.91
O_3/CaO_2	0.075	1.61	$O_3/CaO_2/HCO_3^-$	3.63	1.85

Table S6 Comparison with other techniques on OTC elimination.

Technology	Concentration of OTC (mg/L)	Treatment Time (min)	Kinetic constant (min^{-1})	Removal efficiency (%)	Energy efficiency (g/kWh)	Ref.
Al^0-Gr-Fe^0/O_2	50	60	——	100.0	0.0013	[1]
FBBON/Ag/UCN	10	60	0.034	91.7	0.0120	[2]
PVDF-based piezocatalytic	20	24	0.091	93.1	——	[3]
Co_3O_4/CNTs	20	80	0.034	93.6	——	[4]
Co_3O_4/TiO_2/GO	10	90	0.027	91.0	——	[5]
Cu_2O/α-Fe_2O_3/FTO	10	60	0.021	73.3	——	[6]
$O_3/CaO_2/HCO_3^-$	40	30	4.179	94.4	1.91	This work

References

[1] Y. Liu, Q. Fan, J. Wang, Zn-Fe-CNTs catalytic in situ generation of H_2O_2 for Fenton-like degradation of sulfamethoxazole, J. Hazard. Mater. 342 (2018), 166-176.

[2] J. Ni, D. Liu, W. Wang, A. Wang, J. Jia, J. Tian, Z. Xing, Hierarchical defect-rich flower-like BiOBr/Ag nanoparticles/ultrathing-C_3N_4 with transfer channels plasmonic Z-scheme heterojunction photocatalyst for accelerated visible-light-driven photothermal-photocatalytic oxytetracycline degradation, Chem. Eng. J., 419 (2021), 129969.

[3] W. Ma, B. Yao, W. Zhang, Y. He, Y. Yu, J. Niu, Fabrication of PVDF-based piezocatalytic active membrane with enhanced oxytetracycline degradation efficiency through embedding few-layer E-MoS_2 nanosheets, Chem. Eng. J. 415 (2021), 129000.

[4] D. Liu, M. Li, X. Li, F. Ren, P. Sun, L. Zhou, Core-shell Zn/Co MOFs derived Co_3O_4/CNTs as an efficient magnetic heterogeneous catalyst for persulfate activation and oxytetracycline degradation, Chem. Eng. J. 387 (2020), 124008.

[5] K. Brown, J. Kulis, B. Thomson, T. Chapman, D. Mawhinney, Occurrence of antibiotics in hospital, residential, and dairy effluent, municipal wastewater, and the rio grande in new mexico, Sci. Total Environ. 366 (2006) 772-783.

[6] L. Wang, H. Yang, C. Zhang, Y. Mo, X. Lu, Determination of oxytetracycline, tetracycline and chloramphenicol antibiotics in animal feeds using subcritical water extraction and high performance liquid chromatography, Anal. Chim. Acta. 619 (2008) 54-58.
